# Henoch-Schönlein Purpura-Associated Hemorrhagic Shock After Secondary Norovirus Infection

**DOI:** 10.7759/cureus.11653

**Published:** 2020-11-23

**Authors:** Sara K McLaughlin, Lindsey Lawrence, Jeremy Adler, Hiral Mehta

**Affiliations:** 1 Pediatrics, University of Michigan, Ann Arbor, USA; 2 Pediatric Gastroenterology, University of Michigan, Ann Arbor, USA; 3 Pediatrics, Children's Hospital of Philadelphia, Philadelphia, USA

**Keywords:** hemorrhagic shock, norovirus, henoch schönlein purpura

## Abstract

Henoch-Schönlein purpura (HSP) is a small-vessel vasculitis, typically involving the skin, joints, kidneys, and gastrointestinal (GI) tract. Although GI bleeding with HSP can occur, massive GI hemorrhage is rare. It is well documented that HSP can be triggered by a preceding infection, often of the upper respiratory tract. Infections that occur after the development of HSP and trigger worsening of the disease or new complications have not been well reported. We present the case of a three-year-old previously healthy boy who developed HSP with typical signs and symptoms, including hematochezia that resolved after treatment with intravenous steroids. The patient then contracted norovirus and subsequently developed massive GI bleeding, leading to hemorrhagic shock and requiring admission to an intensive care unit. This case demonstrates that secondary infection, such as norovirus infection, can precipitate worsening of underlying HSP vasculitis and lead to acute clinical decompensation. Clinicians should be aware of the risk of acute clinical changes in patients with HSP.

## Introduction

Henoch-Schönlein purpura (HSP) is the most common systemic vasculitis in children, with an incidence of 10 to 30 per 100,000 children annually [1,2]. The classic presentation includes the tetrad of palpable purpura, arthritis or arthralgias, abdominal pain, and renal involvement [3]. Gastrointestinal (GI) manifestations of HSP occur in the majority of cases, most commonly as abdominal pain or GI bleeding [4]. Massive GI bleeding and hemorrhagic shock are rare, described in select case reports [5-7]. We present a case of HSP in a previously healthy three-year-old boy who demonstrated initial clinical improvement until he contracted norovirus and subsequently developed hemorrhagic shock.

## Case presentation

**Figure 1:**
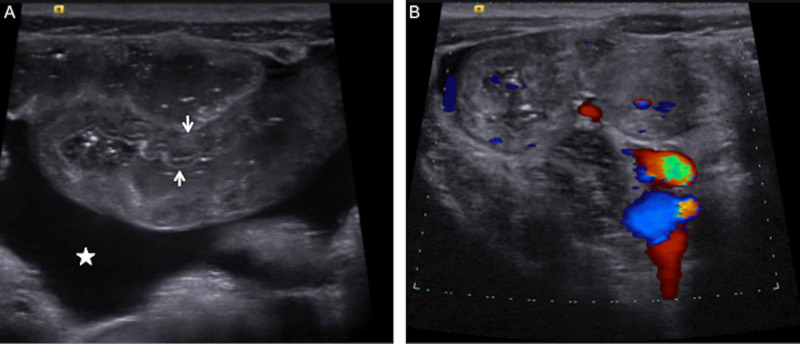
Abdominal Ultrasound A) Lower abdomen midline image demonstrating several loops of small bowel with concentric bowel thickening (between the arrows) and associated simple-appearing ascites (star). B) Right lower quadrant image with color Doppler demonstrating preserved bowel wall flow. Real-time scanning demonstrated no evidence of bowel intussusception or intraperitoneal fluid collection.

A three-year-old unimmunized boy with no significant past medical history developed abdominal pain, nausea, and vomiting. Five days later, he developed arthralgias and a palpable purpuric lower extremity rash; he presented to his pediatrician and was diagnosed with HSP. He was admitted to a local children’s hospital from days six to eight of illness for hydration and pain management. On day 11 of illness, he presented to a different pediatric Emergency Department (ED) with abdominal pain and facial and scrotal edema and was admitted for dehydration. While hospitalized, he experienced multiple episodes of hematochezia, prompting initiation of intravenous (IV) methylprednisolone, 15 mg/kg for two doses. Abdominal ultrasound demonstrated thickened small bowel loops (without intussusception), consistent with HSP (Figure 1).

Hematochezia, edema, abdominal pain, and joint pain resolved after initiation of steroids, though the rash persisted. He was discharged on a month-long prednisone taper. Discharge laboratory studies were notable for leukocytosis, anemia, thrombocytosis, age-appropriate creatinine, hypoalbuminemia, and an elevated urine protein-to-creatinine ratio (UPC) (Table 1).

**Table 1:** Initial Admission Discharge Laboratory Studies

Lab	Value	Reference Range
White blood cell count	18.7 K/uL	6-15 K/uL
Hemoglobin	9.9 g/dL	11-14 g/dL
Hematocrit	29.8%	34-41%
Platelet count	482 K/uL	150-400 K/uL
Sodium	136 mmol/L	132-145 mmol/L
Potassium	4.0 mmol/L	3.3-5.0 mmol/L
Chloride	103 mmol/L	98-108 mmol/L
Bicarbonate	25 mmol/L	18-28 mmol/L
Blood Urea Nitrogen	7 mg/dL	5-20 mg/dL
Creatinine	0.21 mg/dL	0.20-0.60 mg/dL
Glucose	96 mg/dL	50-130 mg/dL
Albumin	3.0 g/dL	3.2-5.2 g/dL
Urine protein-to-creatinine ratio	0.23	0.01-0.18

Three days later, on day 17 of illness, the patient acutely developed abdominal pain, non-bloody emesis, and hematochezia, which progressed to a large volume of bright red blood per rectum, prompting re-presentation to the ED. On arrival, the patient was ashen and unresponsive. Vital signs were notable for heart rate 150 beats per minute and blood pressure 80/50 mmHg. Mucous membranes were dry. Extremities were cool with weak pulses and capillary refill greater than five seconds, consistent with class III-IV hemorrhage and hypovolemic shock. Petechiae and purpura were noted on bilateral lower extremities. Laboratory studies showed marked leukocytosis, thrombocytosis, and uremia reflecting hemoconcentration (Table 2).

**Table 2:** Re-Presentation Initial Laboratory Studies

Lab	Value	Reference Range
White blood cell count	63.6 K/uL	6-15 K/uL
Hemoglobin	12.2 g/dL	11-14 g/dL
Hematocrit	37.2%	34-41%
Platelet count	1,176 K/uL	150-400 K/uL
Sodium	142 mmol/L	132-145 mmol/L
Potassium	4.6 mmol/L	3.3-5.0 mmol/L
Chloride	104 mmol/L	98-108 mmol/L
Bicarbonate	24 mmol/L	18-28 mmol/L
Blood Urea Nitrogen	24 mg/dL	5-20 mg/dL
Creatinine	0.42 mg/dL	0.20-0.60 mg/dL
Glucose	198 mg/dL	50-130 mg/dL
Albumin	3.7 g/dL	3.2-5.2 g/dL
Urine protein-to-creatinine ratio	0.42	0.01-0.18

He was fluid resuscitated with 60 ml/kg normal saline and 10 ml/kg red blood cells. Following resuscitation, laboratory studies were notable for leukocytosis, worsened anemia, thrombocytosis, and worsened hypoalbuminemia (Table 3).

**Table 3:** Post-Resuscitation Laboratory Studies

Lab	Value	Reference Range
White blood cell count	16.3 K/uL	6-15 K/uL
Hemoglobin	8.4 g/dL	11-14 g/dL
Hematocrit	25.5%	34-41%
Platelet count	442 K/uL	150-400 K/uL
Sodium	140 mmol/L	132-145 mmol/L
Potassium	4.4 mmol/L	3.3-5.0 mmol/L
Chloride	110 mmol/L	98-108 mmol/L
Bicarbonate	25 mmol/L	18-28 mmol/L
Blood Urea Nitrogen	10 mg/dL	5-20 mg/dL
Creatinine	0.21 mg/dL	0.20-0.60 mg/dL
Glucose	145 mg/dL	50-130 mg/dL
Albumin	2.7 g/dL	3.2-5.2 g/dL

**Figure 2:**
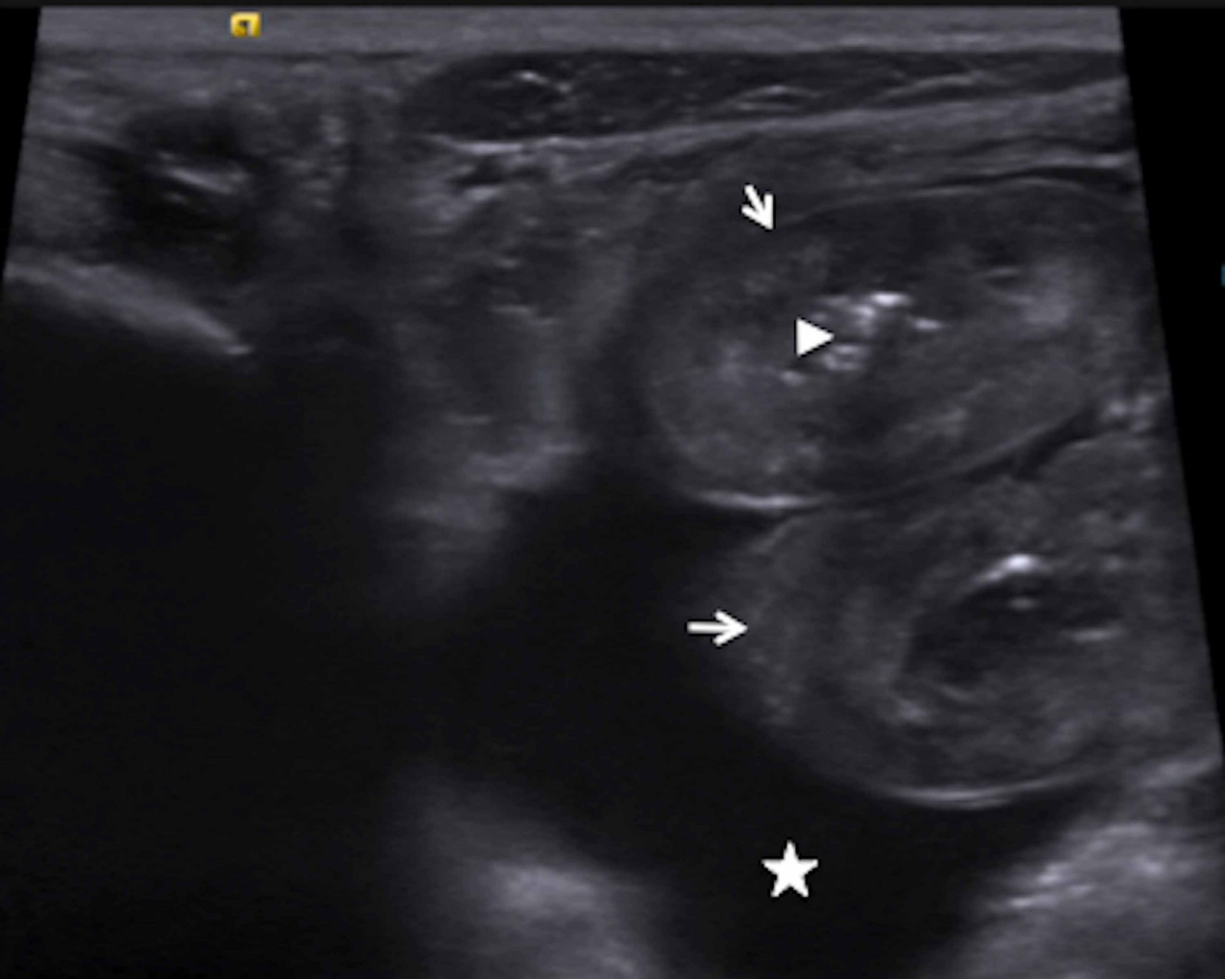
Repeat Abdominal Ultrasound Image of the lower abdomen midline. Again several loops of small bowel with concentric bowel thickening (arrows) and associated simple-appearing ascites (star) are seen. Some bowel loops are fluid-filled, debris-filled and/or gas-filled (arrowhead demonstrates gas in the lumen). Real-time scanning showed no bowel intussusception or intraperitoneal fluid collection.

Abdominal ultrasound revealed dilated loops of edematous bowel, similar to the prior ultrasound, still without signs of intussusception (Figure 2).

The patient was started on IV methylprednisolone and a pantoprazole infusion. Endoscopy was not performed as risks of perforation with hypoalbuminemia and vasculitis outweighed potential benefit. Hypotension resolved and the patient's mental status began improving although he remained lethargic. He was admitted to the pediatric intensive care unit (PICU) for further management of hemorrhagic shock and for close monitoring given continued concerning clinical status.

Given the patient’s unvaccinated status, ill appearance, and leukocytosis raising concern for sepsis, blood and urine cultures were collected and he was started on piperacillin-tazobactam. Cultures came back negative and the antibiotic was discontinued. Gastrointestinal antigen panel, obtained to evaluate for infectious etiologies, was positive for norovirus; additional history-taking revealed that the patient’s siblings had developed typical symptoms of viral gastroenteritis the preceding day. Antineutrophil cytoplasmic antibodies and antistreptolysin O titers were negative. Once started on methylprednisolone and pantoprazole, GI bleeding resolved within 24 hours and hemoglobin stabilized. The patient’s mental status returned to baseline, suggesting initial alteration was due to shock rather than central nervous system HSP involvement. He was transitioned to an oral steroid taper, transferred to a general pediatrics unit, and discharged home eight days following admission. He remained free of GI bleeding and overall symptom-free at follow-up visits, with a normalized UPC. 

## Discussion

HSP is the most common vasculitis in children [8,9]. In addition to the classic tetrad of palpable purpura or petechiae, arthritis or arthralgias, abdominal pain, and renal involvement, HSP also can affect the central nervous system, scrotum, lungs, and GI tract [8]. GI manifestations occur in the majority of patients, most commonly as post-prandial epigastric or periumbilical abdominal pain, similar to bowel angina/ischemia. In fact, earlier HSP literature referred to “bowel angina” as a diagnostic criterion [10]. Intussusception is the most common surgical complication of HSP, with an incidence of 1-14%, presumably due to a lead point from intramural hemorrhage or edema. Other GI complications include perforation, obstruction, stricture, and GI bleeding [11].

GI bleeding occurs in 18-52% of cases of HSP [11] and typically is mild, presenting as melena or hematochezia [4,11]. Although uncommon, cases of severe GI hemorrhage with HSP have been reported. Massive GI bleeding in these cases was attributed to the natural history of HSP [5,7], illness-related stress gastritis [6], or possibly a complication of treatment of the initial disease [12]. No secondary insults (infection, injury, etc.) were reported, although in one case, hemorrhage did not occur until three weeks after HSP diagnosis [6]. Severe GI involvement is more common in adults with HSP, with one study reporting a prevalence of 48%. Half of those with GI involvement had serious GI bleeding, and of those, 11% required transfusion, or surgery, or their bleeding led to death [13].

In this case, the patient acutely developed GI bleeding and hemorrhagic shock on day 17 of illness, despite resolution of mild hematochezia for the preceding four days. These developed after the patient contracted norovirus. Since the introduction of the rotavirus vaccine, norovirus has emerged as the leading cause of medically-attended gastroenteritis in children in the United States [14]. Norovirus commonly presents with vomiting, non-bloody diarrhea, and abdominal pain, but very rarely is associated with GI bleeding [15]. GI pathogens including shigella, salmonella, campylobacter, and, less commonly, norovirus, have been reported to precede the development of HSP [11,16]. To our knowledge, there have not been reports of norovirus infection following a primary HSP diagnosis and triggering a complication or clinical worsening. Additionally, none of the previously-reported cases of GI hemorrhage in HSP described a secondary infection thought to precipitate or exacerbate the bleed.

Our patient’s GI bleed cannot readily be attributed to a more common or previously-described etiology: Although endoscopy was not performed, we do not believe the patient had an underlying gastritis or ulcer. Their natural history is not consistent with the acute onset of bright red blood per rectum that resolves promptly with steroids. Intussusception, a GI complication of HSP, typically presents with pain but can include rectal bleeding; our patient had serial imaging negative for intussusception. Our patient did not have thrombocytopenia or a coagulopathy. Other causes of GI bleeding include vascular malformation, polyp, or Meckel’s diverticulum; none would be expected to resolve with steroid administration. Our patient’s ultrasound imaging did show thick-walled small bowel, as seen in HSP small bowel vasculitis, and bleeding improved with steroids, all consistent with bleeding secondary to HSP vasculitis.

Interestingly, norovirus has been described with hematochezia in patients with inflammatory bowel disease (IBD) who contract the infection [17]. In the general population, norovirus infection alone typically does not cause GI bleeding. In the case of IBD, norovirus is thought to precipitate a disease flare resulting in bleeding, and does not directly cause GI bleeding. Research into mechanisms of norovirus pathology in general is limited due to an inability to culture the virus and a lack of animal models [18]. In our case, the patient developed hemorrhagic shock after contracting norovirus infection while recovering from HSP. It is possible that norovirus caused massive GI bleeding via an increase in the inflammation of perivasculitis. It also is possible that norovirus led to worsening of HSP through another mechanism. Since resolution of HSP and norovirus infection, our patient has been well and has no evidence of IBD. As there was no evidence of alternative causes of GI bleed, we hypothesize that introduction of norovirus to a GI tract inflamed from HSP led to severe bleeding in our patient’s case.

## Conclusions

We describe the case of a three-year-old previously healthy boy who developed HSP. After clinical improvement and resolution of initial hematochezia, the patient contracted norovirus and developed massive GI bleeding, leading to hemorrhagic shock requiring transfusion and PICU admission. To our knowledge, GI viral infection in the setting of preceding HSP and leading to massive GI bleed has not been reported previously. Clinicians should be aware of the risk of severe late GI bleeds in patients with HSP and provide anticipatory guidance to families as well, as these complications can be acutely life-threatening and require immediate medical attention, as in our patient’s case.
